# Identification of Pathogen Causing Bulb Rot in *Fritillaria taipaiensis* P. Y. Li and Establishment of Detection Methods

**DOI:** 10.3390/plants13162236

**Published:** 2024-08-12

**Authors:** Shijie Wang, Keke Chen, Jiaqi Guo, Panwang Zhang, Yuchen Li, Zhenghao Xu, Langjun Cui, Yi Qiang

**Affiliations:** 1 National Engineering Laboratory for Resource Development of Endangered Chinese Crude Drugs in Northwest of China, Shaanxi Normal University, Xi’an 710119, China; wangshijie3586@snnu.edu.cn (S.W.); guojiaqi2@snnu.edu.cn (J.G.); zerevis@snnu.edu.cn (P.Z.); 19992314679@snnu.edu.cn (Y.L.); zhxu@snnu.edu.cn (Z.X.); 2 Key Laboratory of Medicinal Resources and Natural Pharmaceutical Chemistry, The Ministry of Education, Shaanxi Normal University, Xi’an 710119, China; 3 College of Life Sciences, Shaanxi Normal University, Xi’an 710119, China; 4 School of Biological and Environmental Engineering, Xi’an University, Xi’an 710065, China; kkchen1125@foxmail.com

**Keywords:** *Fritillaria taipaiensis* P. Y. Li, *Fusarium solani*, loop-mediated isothermal amplification, qPCR

## Abstract

*Fritillaria taipaiensis* P. Y. Li (*F. taipaiensis*) is a traditional Chinese herbal medicine that has been used for over two millennia to treat cough and expectoration. However, the increasing cultivation of *F. taipaiensis* has led to the spread of bulb rot diseases. In this study, pathogens were isolated from rotten *F. taipaiensis* bulbs. Through molecular identification, pathogenicity testing, morphological assessment, and microscopy, *Fusarium solani* was identified as the pathogen causing bulb rot in *F. taipaiensis*. The colonization of *F. solani* in the bulbs was investigated through microscopic observation. The rapid and accurate detection of this pathogen will contribute to better disease monitoring and control. Loop-mediated isothermal amplification (LAMP) and qPCR methods were established to quickly and specifically identify this pathogen. These results provide valuable insights for further research on the prediction, rapid detection, and effective prevention and control of bulb rot in *F. taipaiensis*.

## 1. Introduction

*Fritillaria taipaiensis* P. Y. Li (*F. taipaiensis*) is a perennial herbaceous plant belonging to the *Fritillaria* genus within the Liliaceae family. It has been cultivated as a herbal medicine in China for over two millennia [1,2]. *F. taipaiensis* is a prominent variety used as *Fritillaria cirrhosa* D. Don in traditional Chinese medicine. The medicinal portion of the plant is the bulb, and the principal active component is sipeimine, which belongs to the class of steroid alkaloids [3]. The distinctive aromatic ring structure of sipeimine gives the bulb its medicinal value and helps it to exert curative effects involving lung moisturization, cough relief, and phlegm elimination [4,5]. The plant is widely distributed in Shaanxi, Sichuan, and other provinces [6]. As a valuable traditional Chinese medicine, its wild resources are scarce. In order to meet the demands of the market, it is cultivated primarily through artificial means.

However, as the planting area and continuous cultivation expand, the occurrence of diseases has become increasingly severe in recent years. Among these diseases, bulb rot is one of the most impactful, posing a significant threat to the *F. taipaiensis* industry [7]. *F. taipaiensis* can reproduce asexually through bulbs, and bulbs infected with pathogens often become the main potential source of infection, leading to the broad spread of diseases to whole new fields. Although the use of fungicides is effective in controlling diseases, fungicide residues and pathogens that produce mycotoxins that are toxic to humans can affect the quality of *F. taipaiensis* [8,9,10]. Therefore, in order to minimize the losses caused by diseases, cultivation management and the technological development of disease detection and prevention are crucial.

With regard to its diagnosis, molecular diagnostic techniques, including PCR, quantitative real-time PCR (qPCR), loop-mediated isothermal amplification (LAMP), recombinase polymerase amplification (RPA), rolling circle amplification (RCA), and nucleic acid sequence-based amplification (NASBA) are undergoing constant development [11,12]. Among these techniques, LAMP operates at a constant temperature without the need for a thermal cycler, electrophoresis, or a gel imaging system. This technique is highly accurate and specific and allows for the visualization and analysis of LAMP reaction products through combinations of metal ion indicators or DNA-intercalating dyes, which has been proven effective in field disease control [13,14,15,16,17,18]. Meanwhile, qPCR employs species-specific primers to simultaneously identify and quantify plant pathogens. It has already been applied to some extent in monitoring pathogens in plants and soil [19,20,21,22,23].

*Fusarium* is a species-rich genus and is arguably the most important group of mycotoxigenic plant pathogens [24,25,26]. The internal transcribed spacer region (ITS rDNA) has been selected as the standard barcode site for fungi [27]. However, this region and the 5′ end of the nuclear ribosomal large subunit (*28S rDNA*) are typically too conserved to distinguish *Fusarium* species boundaries [28]. The translation elongation factor 1-α (*EF-1α*) gene is typically employed for the identification of *Fusarium* isolates at or near the species level [29,30]. The ATP-citrate lyase 1 (*acl1*) gene has also been employed to investigate the phylogenetic relationships between various species of *Fusarium* and other members of the family Nectriaceae [31,32].

This study identified *Fusarium solani* as the pathogen causing bulb rot in *F. taipaiensis* through microscopic, morphological, and molecular characteristic analyses and through pathogenicity testing. Microscopic observations were conducted on both healthy bulbs and bulbs inoculated with *F. solani*. Finally, two diagnostic schemes based on LAMP and qPCR were developed to accurately detect *F. solani* in infected plant tissues, thus enabling early management and control of the disease affecting *F. taipaiensis*.

## 2. Results

### 2.1. Morphological Characteristics and Molecular Identification of Isolate FTA1

**Figure 1:**
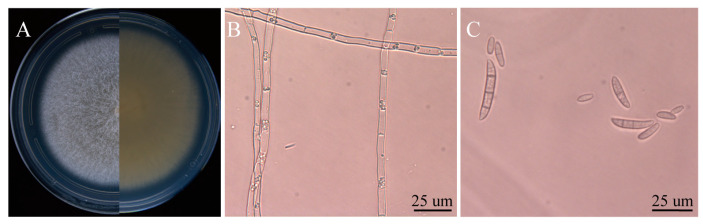
The morphological characteristics of FTA1. (**A**) The FTA1 phenotype following a 5-day culture period on potato dextrose agar (PDA). (**B**) The hyphal morphology of FTA1. (**C**) The morphological characteristics of FTA1’s microconidia, macroconidia, and conidia. Scale bar: 25 μm.

**Figure 2:**
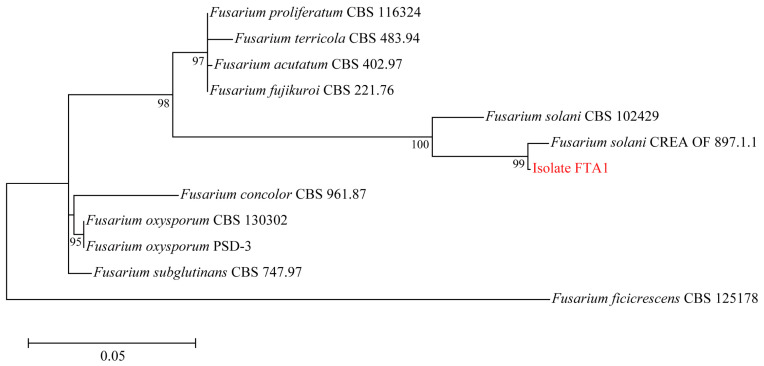
Maximum likelihood phylogenetic tree constructed using the concatenated sequence data (ITS, *EF-1α*, and *ACT*) from FTA1 and the related *Fusarium* species. Bootstrap support values for maximum likelihood greater than 70% are indicated.

**Figure 3:**
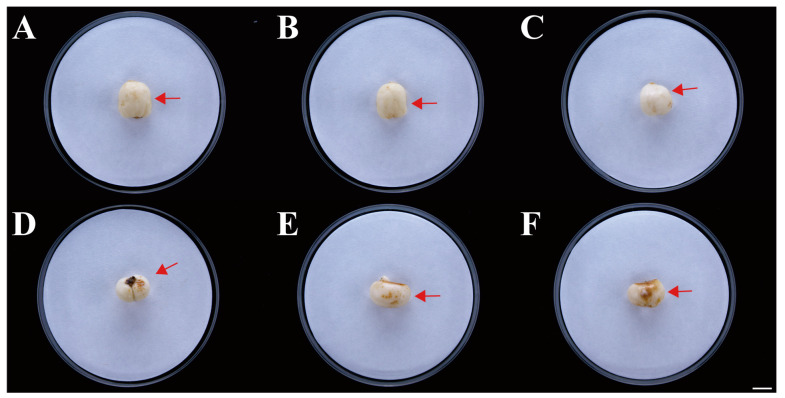
Symptoms of *F. taipaiensis* inoculated with FTA1. (**A**–**C**) No symptoms of *F. taipaiensis* artificial infection with potato dextrose broth (PDB). The arrow points to the location where PDB was inoculated. (**D**–**F**) Distinct brown disease spots appeared after artificial inoculation with FTA1. The arrow points to the location where FTA1 was inoculated. Scale bar: 1 cm.

Six isolates with similar morphological characteristics were obtained, and the representative isolate FTA1 was used for further analysis. The isolate FTA1 grew well on potato dextrose agar (PDA) (200 g potato extracts/L, 2% glucose, and 2% agar) medium, forming circular colonies with white aerial hyphae in the early stage. Later, a white flocculent colony was observed, with its underside appearing dull yellow (Figure 1A). Microscopically, septate hyphae (Figure 1B), small, elliptical conidia, and typical falcate macroconidia with 1–3 transverse septa were observed (Figure 1C). The ITS, *EF-1α*, and actin (*ACT*) sequences of FTA1 were deposited in GenBank with the accession numbers PP758636, PP766248, and PP785370, respectively. The multigene phylogenetic analysis revealed that isolate FTA1 and *F. solani* CREA OF 897.1.1 (OQ255944, OQ274879, and OQ249626) clustered within the same clade with 99% bootstrap support (Figure 2). It was speculated that this strain is *F. solani*. Five days after inoculation, no significant changes were observed in *F. taipaiensis* bulbs inoculated with potato dextrose broth (PDB) (200 g potato extracts/L and 2% glucose) medium, while obvious decay symptoms occurred in *F. taipaiensis* bulbs inoculated with FTA1 (Figure 3). There were yellowish-brown spots on the surfaces of *F. taipaiensis* bulbs inoculated with the FTA1 strain, which were similar to the decay symptoms of *F. taipaiensis* bulbs in the field (Figure S1). The pathogen was re-isolated from the inoculated bulbs and identified as *F. solani* via molecular and morphological methods that conformed to Koch’s postulates.

### 2.2. Microscopic Observation of Pathogenic Infection on Bulbs of F. taipaiensis

**Figure 4:**
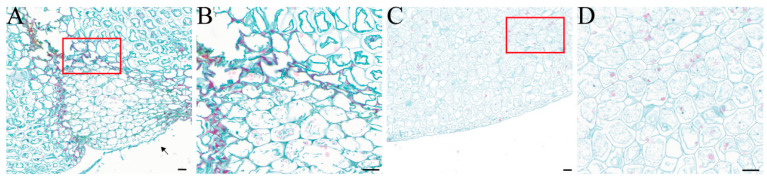
Observation of the longitudinal anatomical structure of healthy bulbs and diseased bulbs after artificial inoculation with FTA1, utilizing Safranin O-Fast Green staining. (**A**) Diseased bulb; the arrow points to the location of the FTA1 spore suspension inoculation. (**B**) Partial magnified view of the diseased bulb. (**C**) Healthy bulb treated with PDB. (**D**) Partial magnified view of the healthy bulb. Scale bar: 100 μm.

Under microscopic observation, *F. solani* was found to colonize the surface of the *F. taipaiensis* bulbs. The healthy bulb tissue was structurally intact, with full-shaped cells containing starch grains. It was observed that the invading fungi gradually invaded the bulb interior in the form of hyphae and spores. The cell walls of infected cells were stained red, showing lignification and thickening; in severely infected cells, the cell membranes ruptured, and their organelles, starch grains, and other inclusions disappeared (Figure 4).

**Figure 5:**
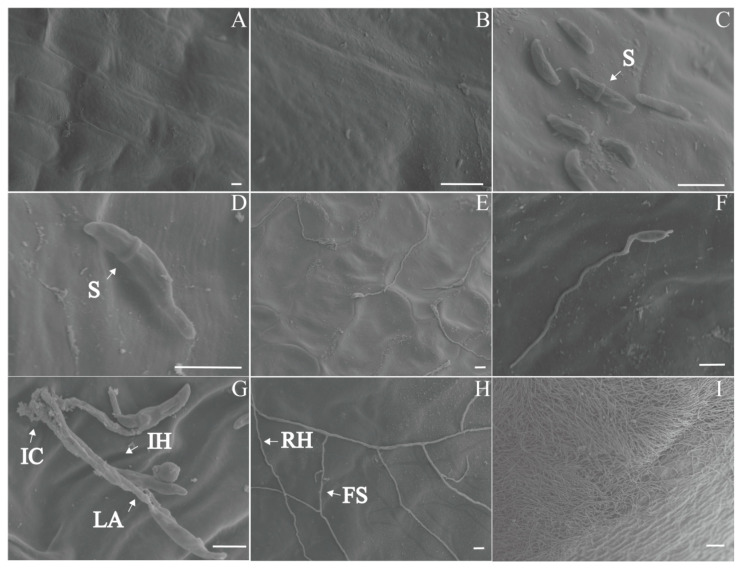
Scanning electron microscope (SEM) micro-observations of different stages of bulb rot disease in *F. taipaiensis*. (**A**) Surface of a healthy bulb. (**B**) Magnified image of the healthy bulb surface. (**C**) Bulb surface 6 h after inoculation. (**D**) Conidia began to germinate on the bulb surface 6 h after inoculation. (**E**) Bulb surface 12 h after inoculation. (**F**) Conidia germinated on the bulb surface, forming short hyphae 12 h after inoculation. (**G**) Infection cushions and a lobate appressorium formed on the surface of the bulb 1 day after inoculation. (**H**) A hyphal network formed on the surface of the bulb 2 days after inoculation. (**I**) A dense hyphal network formed on the surface of the bulb 3–4 days after inoculation. Scale bar: (**A**–**H**): 10 μm, (**I**): 100 μm. Abbreviations: **FS**, foot structure; **IC**, infection cushion; **LA**, lobate appressorium; **IH**, infection hypha; **RH**, runner hypha; **S**, septum.

The scanning electron microscope (SEM) examination of healthy bulbs showed that the bulbs had smooth surfaces, intact cell walls, and neatly arranged cells, with no hyphae (Figure 5A). Six hours after inoculation, scythe-shaped conidia colonized in the cell gaps on the bulb surface and started to germinate (Figure 5D). At 12 h, the conidia germinated, and hyphae spread on the surface. Short infectious hyphae indicated direct penetration (Figure 5E). At 24 h, leaf-like appressoria and infection cushions formed (Figure 5G). After 2 dpi, the hyphae branched frequently, forming foot structures (Figure 5H). The hyphae grew into homogenous hyphal networks. After 3-4 dpi, the bulb surface was covered with aerial hyphae (Figure 5I). The colonized areas lost their smoothness, with unclear cell outlines and loose cell arrangements.

### 2.3. Pot Experiment

**Figure 6:**
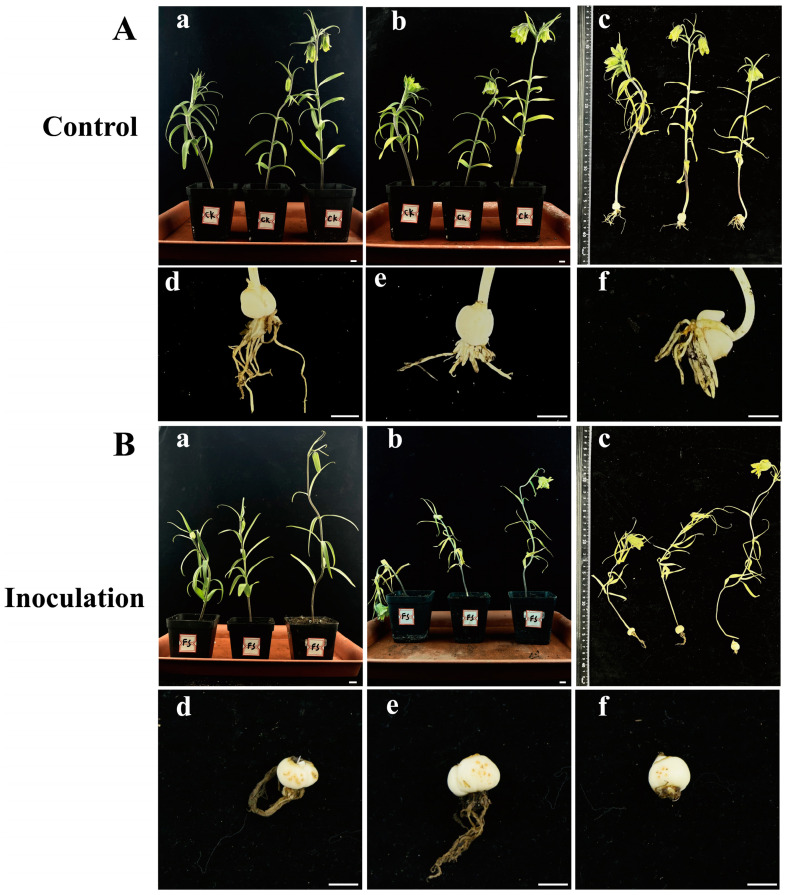
(**A**), (**a**–**c**) Symptoms of *F. taipaiensis* plants at 7, 14, and 21 days after treatment with PDB, and (**d**–**f**) local symptoms of *F. taipaiensis* bulbs at 21 days after treatment with PDB. (**B**), (**a**–**c**) Symptoms of *F. taipaiensis* plants at 7, 14, and 21 days post-inoculation with FTA1. (**d**–**f**) Local symptoms of *F. taipaiensis* bulbs at 21 days post-inoculation with FTA1. Scale bar: 1 cm.

Healthy *F. taipaiensis* plants inoculated with an *F. solani* spore suspension exhibited death or severe wilting symptoms, while the control group showed no significant changes (Figure 6). Fourteen days after inoculation with *F. solani*, the plants in the experimental group exhibited wilting symptoms (Figure 6B), while no obvious symptoms were observed in the aerial parts of the control group (Figure 6A). Twenty-one days after inoculation, the underground parts of the experimental group showed obvious rot, the roots turned brown, and yellowish-brown spots appeared on the bulbs (Figure 6B). The roots of the control group were healthy white, and the bulbs showed no significant symptoms (Figure 6A). The pathogens were re-isolated from the inoculated bulbs and identified as *F. solani* through molecular and morphological methods, conforming to Koch’s postulates. *F. solani* can cause rot in the bulbs of *F. taipaiensis* in the field, posing a significant hazard to the cultivation and production of *F. taipaiensis*. Due to the time-consuming culture purification techniques and expert knowledge of fungal morphology required, it is difficult to rapidly and accurately distinguish fungi. Therefore, it is necessary to develop molecular detection methods to identify this pathogenic fungus.

### 2.4. LAMP and qPCR Primer Selection

An analysis of the *acl1* gene in *Fusarium* species revealed sufficient variability to enable the design of specific primers for *F. solani* (Figure S2). Consequently, the *acl1* gene sequence (GenBank: MW810996) was selected to develop LAMP primers for *F. solani* (Figure S3). To avoid non-specific amplification and ensure detection sensitivity, the LF primer was omitted. Primer sets for LAMP detection based on these genes were designed and are listed in Table 2.

In order to enhance the monitoring and prevention of pathogen infection in the bulb of *F. taipaiensis*, we established a qPCR detection method based on the acl1 gene sequence (GenBank: MW810996). The qPCR primers were designed at specific loci (Figure S4). A standard curve was constructed for *F. solani* using known concentrations of the pathogenic fungus. The standard curve exhibited high reproducibility, enabling the generation of highly specific, sensitive, and reproducible data. The standard curve was constructed based on a diluted standard template, and the number of target genes in unknown samples was then determined by means of interpolation.

### 2.5. Specificity of LAMP and qPCR Assays

**Figure 7:**
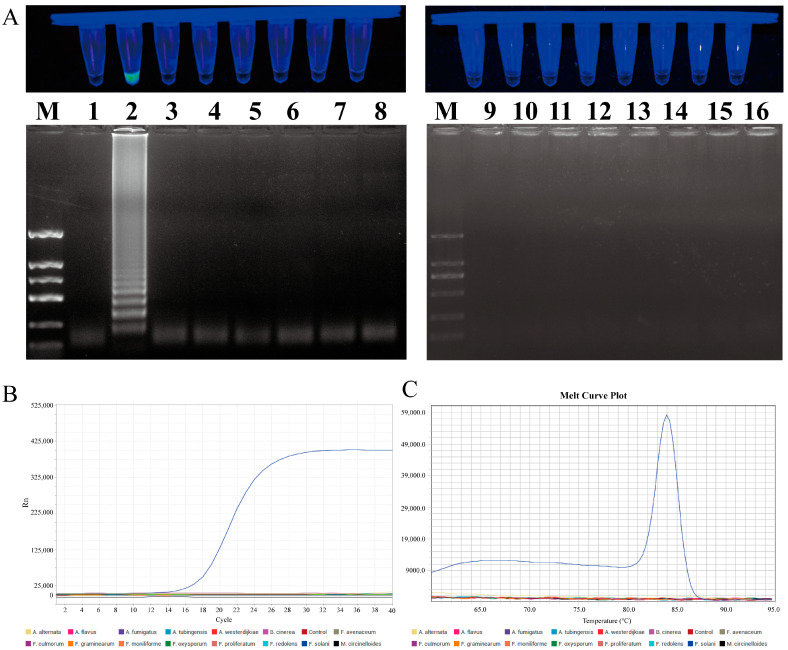
Specificity testing of LAMP and qPCR primers designed for *F. solani*. (**A**) LAMP primer set for *F. solani*. Lane M: DNA marker 2 k; Lanes 1 to 16: ddH_2_O, *F. solani*, *F. avenaceum*, *F. oxysporum*, *F. proliferatum*, *F. redolens*, *F. graminearum*, *F. culmorum*, *F. moniliforme*, *Alternaria alternata*, *Botrytis cinerea*, *A. fumigatus*, *A. tubingensis*, *A. flavus*, *A. westerdijkiae*, and *Mucor circinelloides*. (**B**) Amplification curve for qPCR primer specificity detection. (**C**) Melting peaks for qPCR primer specificity detection.

In order to evaluate the specificity of the LAMP and qPCR primers, a series of tests was conducted on eight different *Fusarium* species and seven species from other genera. In the LAMP primer set specificity test, only the *F. solani* reaction mixture produced a positive result, while the control group and other fungal samples were negative. The 1% agarose gel electrophoresis result was consistent with the previous findings, with the specific ladder-like bands only amplified in *F. solani*. In the qPCR primer specificity test, only *F. solani* exhibited an amplification curve and a single melting peak, whereas no amplification was observed in the other strains or the control group (Figure 7).

### 2.6. Sensitivity of LAMP Primer Set

**Figure 8:**
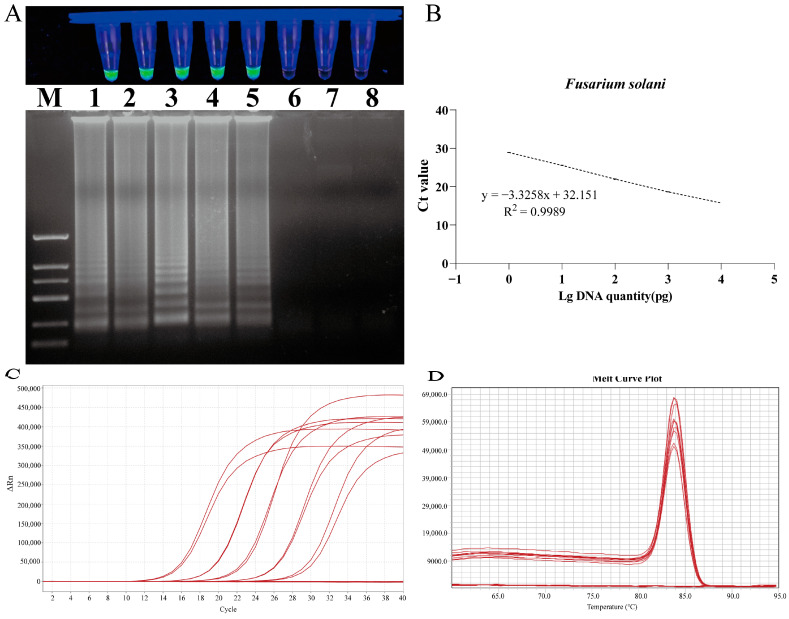
Sensitivity test of LAMP and qPCR primers designed for *F. solani*. (**A**) LAMP primer set for *F. solani*. Lane M: DNA marker 2 k; Lanes 1 to 8: 10 ng/µL, 1 ng/µL, 100 pg/µL, 10 pg/µL, 1 pg/µL, 100 fg/µL, 10 fg/µL, and 1 fg/uL. (**B**) Standard curve of specific qPCR for *F. solani*. (**C**) Amplification curves for qPCR primer sensitivity detection. (**D**) Melting peaks for qPCR primer sensitivity detection.

The detection limit of the LAMP assay was evaluated by employing serial dilutions of DNA samples from *F. solani* (Figure 8). The sensitivity results for *F. solani* revealed that the reaction mixture containing 1 pg of DNA tested positive, while the mixture containing 100 fg of DNA tested negative. The amplified products from each LAMP assay were subjected to 1% agarose gel electrophoresis, which revealed the presence of continuous DNA bands in each positive result (Figure 8).

### 2.7. Establishment of Absolute Quantitative Standard Curve for qPCR

A quantitative detection method was established based on the gradient concentration of *F. solani* DNA and the cycle threshold (Ct) value of qPCR reactions. A standard curve of the target gene was generated through linear regression analysis by continuously diluting each target gene 10-fold. The quantitative cycle (Cq) value was plotted on the y-axis against the logarithm of the initial DNA dilution on the x-axis. The results demonstrated a robust linear relationship between the Ct value and the DNA concentration of *F. solani* in the range of 1.0 × 10^4^ to 1.0 × 10^0^ pg/μL. The linear equation was y = −3.3258x + 32.151, the correlation coefficient was R^2^ = 0.9989, and the amplification efficiency was E = 99.84%. The melting temperature of the amplification products was 83.934 ± 0.1 °C, indicating good specificity (Figure 8).

### 2.8. Application of LAMP and qPCR-Based Diagnostic Methods

**Figure 9:**
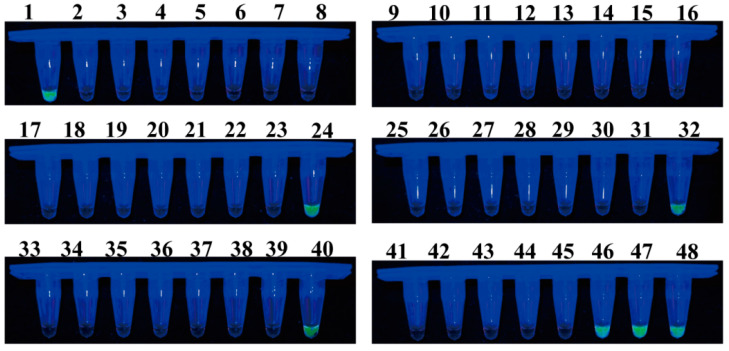
Application of the LAMP assay for the detection of *F. solani* in 48 bulbs of *F. taipaiensis*. Samples emitting bright green fluorescence were evaluated as positive, while non-fluorescing samples were evaluated as negative.

A total of 48 samples of healthy or infected individuals were randomly collected in Guangyuan, Sichuan Province, Hanzhong, Shaanxi Province, and Shangluo, Shaanxi Province, to evaluate the effectiveness of LAMP and qPCR in detecting *F. solani* in the bulbs of *F. taipaiensis*. In the LAMP detection system, 7 of the 48 samples tested positive (Figure 9). The qPCR detection system yielded identical results to the LAMP system, with a detected DNA concentration range of 3.58–985.84 pg (Table 1). Moreover, additional samples, including those that tested positive, were utilized for pathogen isolation. Among these samples, the anticipated *F. solani* was successfully isolated and identified through a combination of morphological and molecular biological analyses.

## 3. Discussion

A study based in Shaanxi Province, China, reported that the pathogen causing bulb rot disease in *F. taipaiensis* is *F. oxysporum* [7]. However, in this study, the pathogenic fungus isolated from infected bulbs in a field in Sichuan Province, China, was identified as *F. solani*. This is the first report of *F. solani* infecting *F. taipaiensis. F. solani* can cause diseases in various fruit plants, such as banana, papaya, and pineapple, as it produces chlamydospores that can persist in soil and plant debris for a long time, serving as a source of the inoculum [33,34,35,36,37,38]. Therefore, exploring the invasion and colonization patterns of *F. solani* and establishing appropriate detection methods are crucial for the management and control of disease in the *F. taipaiensis* industry.

This study found that *F. taipaiensis* bulbs have different epidermal cell shapes, obvious intercellular spaces, and thin cell walls, which easily lead to mechanical wounds during contact and friction between the bulbs’ epidermis and external hard substances in the growth process of *F. taipaiensis*, thus promoting the invasion of pathogens. *F. solani* infects the bulb of *F. taipaiensis* through hyphae and spores on the surface, subsequently invading the interior. Infected cells exhibit phenomena, such as cytoplasmic concentration, cell deformation, and a reduction in or even the disappearance of organelles and starch granules. *F. solani* infection in bulbs can be divided into three stages: Stage I: the initial colonization stage, *F. solani* germinates within 6–12 h, and short infectious hyphae grow and spread on the surface; Stage II: the main infection stage, the runner hyphae begin to branch at a high frequency, forming foot structures, a lobate appressorium, and infection cushions; Stage III, the final infection stage, the entire tissue necrotizes, and the surface is covered with aerial hyphae. This is similar to the process of *Fusarium* species infection in other plants [39,40,41,42].

The destruction of host tissues by fungi is not only due to the mechanical action of growth and infection of cells but also possibly related to the metabolites that the fungi produce [43]. The cell-wall-degrading enzymes from *Fusarium* decompose suberin, and some toxins produced by *Fusarium* species can facilitate their infection and colonization process [44,45]. This indicates that *F. solani* can colonize the surface of healthy bulbs, and the toxins that it produces, in addition to facilitating its infection, are highly harmful to humans, significantly affecting the quality of *F. taipaiensis*. Therefore, this study established two detection methods to provide a strategy for the early detection and prevention of bulb rot in *F. taipaiensis*.

The most crucial step in PCR-based molecular diagnostic methods is primer design. Rational selection of the target gene is important as it directly affects the specificity and sensitivity of LAMP and qPCR detection [46]. Among the published LAMP primers for *Fusarium* species, *EF-1α* [47], Secreted in Xylem Genes [48,49], *CYP51C* [50], the intergenic spacer (*IGS*) region of the rRNA genes [51], and *acl1* [52] are commonly used for primer development. The *acl1* gene has also been reported to provide sufficient signals for the phylogenetic analysis of *Fusarium* species complexes [31]. Therefore, in this study, the *acl1* gene was used to design LAMP primers and qPCR primers, and their specificity was proven.

Utilizing two detection methods based on LAMP and qPCR, we investigated *F. solani* contamination in bulbs of *F. taipaiensis* in the field. The detection rate of *F. solani* was 14.6%, and the results of the two methods were identical. We successfully isolated *F. solani* from the positive tested bulbs. The LAMP detection method can be performed without the need for grid power through the use of a portable battery-driven thermal cycler; furthermore, sample preparation and ready-to-use LAMP reaction mixtures do not require skilled personnel or specific laboratory equipment [53]. LAMP technology combined with rapid sample extraction methods can enable rapid disease diagnosis in the field [54,55]. qPCR can be used to quantitatively analyze *F. solani* colonization in bulbs, which is conducive to the more accurate long-term monitoring of disease situations, and this has been applied in some plants [56,57,58]. All of these points demonstrate the tremendous potential of our two developed detection methods for wider use in this field.

Diseases, such as pneumonia, bronchitis, and influenza, can all be treated with the bulbs of *F. taipaiensis*. As the cultivation area of *F. taipaiensis* expands and continuous cultivation increases, the problem of diseases affecting the bulbs in artificial cultivation has become increasingly serious. By combining LAMP and qPCR methods, we can gain a more comprehensive understanding of the presence of pathogenic fungi in the bulbs of *F. taipaiensis*. The LAMP method provides a rapid and intuitive initial judgment, while the qPCR method provides accurate and quantitative results. The combined use of these two methods will help us to more accurately assess the health status of *F. taipaiensis* and provide a scientific basis for subsequent disease prevention and control.

## 4. Materials and Methods

### 4.1. Pathogen Isolation and Identification

In June 2023, diseased tissue was collected from Guangyuan, Sichuan, China (32.52° N, 106.16° E, altitude 1522 m). Tissues were removed from the symptomatic bulbs, surface-sterilized with 75% alcohol for 5 min and 2% sodium hypochlorite for 2 min, and finally rinsed thrice with sterilized distilled water [59]. The tissues were placed on a new solid PDA medium and incubated at 28 °C in the dark. After 3–5 days of cultivation, the mycelia grown from the diseased tissues were isolated and purified. The morphology of the fungal hyphae and spores was observed under a microscope to obtain the morphological characteristics of each stage. The isolated strains were identified using their ITS, *EF-1α*, or *ACT* gene sequences. The fungal genomic DNA was extracted using a fungal genomic DNA extraction kit (Beijing Solarbio Science & Technology Co., Ltd., Beijing, China). PCR was performed using the primers ITS1/ITS4, EF-1α F/EF-1α R, and ACT-512F/ACT-783R (Table 2) [29,60,61], and the products were sequenced by Shanghai Sangon Biotech Co., Ltd. After identification, the following tests were performed on the isolates according to Koch’s postulates. The healthy bulbs of *F. taipaiensis* were sterilized with 75% alcohol for 5 min and 2% NaClO for 2 min and then rinsed three times with sterile water. Then, a 10 μL suspension (1 × 10^6^ conidia/mL) of purified fungi was inoculated onto the bulbs to observe its pathogenicity. The control group was inoculated with 10 uL of PDB medium. Each group contained three replicates. All bulbs were placed in a 25 °C incubator in the dark. Five days after inoculation, the disease symptoms were observed, and the pathogens were re-isolated from the infected tissue and re-identified by sequencing the PCR fragments of ITS, *EF-1α*, and *ACT*; the sequences were then submitted to the National Center for Biotechnology Information (NCBI) GenBank. Based on the sequences of multiple gene combinations, a phylogenetic tree was constructed using the maximum likelihood (ML) method in MEGA11.0 (Table S1).

### 4.2. Microscopic Observation of Pathogen Infection on Bulbs of F. taipaiensis

*F. taipaiensis* bulbs at 3–4 days post-inoculation and healthy bulbs treated with PDB for the same duration were vertically sliced into 1 cm^2^ sections, which were stained in Safranin solution for 2 h and then in Fast Green solution for 20 s. These sections were prepared using a Leica RM2016 automatic vibrating microtome (Leica, Wetzlar, Germany); the sections were then observed under a Nikon Eclipse E100 optical microscope (Nikon, Tokyo, Japan), and images were captured.

Using the same method, healthy bulbs were sterilized and inoculated with the pathogen. The SEM sampling was performed at 0, 6, 12, 24, 48, 72, and 96 h. The bulbs inoculated with pathogenic fungi and the healthy bulbs were washed three times with 0.1 M phosphate buffered saline (PBS) at pH 7.4. The samples were then fixed using 3% glutaraldehyde, post-fixed using 1% osmium tetroxide, and dehydrated using gradient ethanol. The samples were glued to sample holders with conductive adhesive, and the holders were placed into an ion sputterer for spray treatment. The samples were observed using a Hitachi S-3400N scanning electron microscope (Hitachi, Tokyo, Japan).

### 4.3. Pot Experiment

Nutrient soil, vermiculite, and perlite were thoroughly mixed in a ratio of 3:1:1 (v:v:v) and sterilized for 45 min. Healthy four-year-old *F. taipaiensis* plants were placed in a 20 °C incubator for 7 days to adapt to the environment under 12 h light/dark cycles. A 20 mL conidial suspension (1 × 10^6^ conidia/mL) was added to the soil around the base of the plants, while the control group was inoculated with 20 mL of PDB. For each group, at least three healthy plants were inoculated with the conidial suspension. All the plants were placed in a 20 °C incubator with a 12 h light/dark cycle and maintained at 80% relative humidity. The symptoms were checked and photographed.

### 4.4. Design of LAMP and qPCR Primers

Species-specific LAMP primers and qPCR primers for *F. solani* were developed based on the *acl1* gene (Table 2). The gene sequences of closely related species were obtained from NCBI for alignment to identify specific loci. PrimerExplorer V5 software (https://primerexplorer.jp, accessed on 9 October 2023) was used to create the LAMP primers based on the alignment results. These primers included two outer primers (F3, B3), two inner primers (Forward Inner Primer FIP: FIc+F2, Backward Inner Primer BIP: BIc+B2), and a loop primer (Loop Backward Primer (LB)). Based on the DNA sequences within these specific loci, real-time PCR (qPCR) primers were designed using an online primer design tool (https://www.ncbi.nlm.nih.gov/tools/primer-blast/, accessed on 22 October 2023) [62].

### 4.5. Reaction Systems

All primers were synthesized by Sangon Biotech (Shanghai) Co., Ltd. The reaction mixture was prepared according to the method in a previous paper, with some modifications [63]. The LAMP reaction was performed in a 15 μL reaction volume containing 2.5 μL of 10× isothermal amplification buffer (200 mM Tris-HCl pH 8.8, 100 mM KCl, 100 mM (NH_4_)_2_SO_4_, 20 mM MgSO_4_, and 1% Triton X-100), 1.6 mmol/L deoxynucleotide triphosphates (dNTPs), 0.2 μmol/L of each outer primer (F3 and B3), 0.4 μmol/L of each loop primer (LF and LB), 0.8 μmol/L of each inner primer (FIP and BIP), 8 U Bst DNA polymerase (Sangon Biotech Co., Ltd., Shanghai, China), and 1 μL of DNA template. The LAMP reaction mixture for *F. solani* was heated at 65 °C for 60 min, and the reaction was terminated via heating at 85 °C for 5 min. Next, we added 1 µL of SYBR Green I (1000×) to each reaction tube. The color of the solution was observed visually under blue light (470 nm), with positive results emitting a bright green fluorescence and negative results remaining non-fluorescent.

The real-time quantitative PCR (qPCR) was performed in a 20 μL reaction mixture containing 2 μL of template, 0.5 μL each of the forward and reverse primers, 8 μL of double-distilled water, and 10 μL of SYBR Premix Ex Taq mixture (Vazyme Biotech, Nanjing, China). The qPCR was conducted on a QuantStudio™ 3 (Thermo Fisher Scientific, Wilmington, MA, USA). The qPCR analysis for each sample was repeated at least three times. All Ct values were inputted into Graphpad Prism 9 (GraphPad Software, Boston, MA, USA) to calculate the average Ct value and generate a line graph. The qPCR protocol was established as follows: an initial denaturation step at 95 °C for 3 min, followed by 40 cycles of 95 °C for 10 s, and an annealing temperature of 62 °C for 30 s. Afterward, amplification curves and melting curve analyses were performed to confirm that the primers amplified only a single product.

### 4.6. Detection of LAMP Specificity and Sensitivity

The isolate FTA1 and other closely related strains (Table S2) were cultured on PDA solid medium. DNA was then extracted from them and further used to test the primer specificity. The strains used for qPCR specificity detection were the same as those used for the LAMP method. The DNA of FTA1 was diluted with double-distilled water to concentrations of 10 ng/µL, 1 ng/µL, 100 pg/µL, 10 pg/µL, 1 pg/µL, 100 fg/µL, 10 fg/µL, and 1 fg/µL, which served as templates for the sensitivity test. The DNA concentrations were measured using a NanoDrop 2000 (Thermo Scientific, Wilmington, NC, USA). Following this, LAMP detection was performed. Both the specificity and sensitivity tests were performed three times.

### 4.7. Establishment of Absolute Quantitative Standard Curve for qPCR

Based on the gradient concentrations of pathogenic DNA and Ct values of the qPCR reactions, an absolute quantitative detection method was established. An absolute quantitative standard curve was constructed for the primers with good specificity. Fluorescence signals were collected during the annealing phase of each PCR cycle, and the instrument’s software was used for data acquisition and analysis to automatically detect the Ct value of each reaction. After the concentration of mycelial DNA was determined, it was diluted to 8 consecutive concentrations for detection: 10 ng/µL, 1 ng/µL, 100 pg/µL, 10 pg/µL, 1 pg/µL, 100 fg/µL, 10 fg/µL, and 1 fg/µL. After the Ct values corresponding to the DNA concentrations were obtained, a standard curve was generated by using the logarithm of the DNA concentration as the x-axis and the Ct value as the y-axis. Each reaction was repeated three times.

### 4.8. Applications Based on LAMP and qPCR Detection Methods

A total of 48 bulbs that were either healthy or had disease spots were collected in Guangyuan City, Sichuan Province; Hanzhong City, Shaanxi Province; and Shangluo City, Shaanxi Province. A commercial fungal genome extraction kit (Beijing Solarbio Science & Technology Co., Ltd.) was utilized to extract the DNA from the bulbs of *F. taipaiensis*. The samples were then subjected to the LAMP and qPCR methods to detect pathogens. The results of the LAMP method were determined by observing the color change in the solution under blue light (470 nm). The qPCR method employed a linear regression approach to predict the concentration of pathogen DNA detected from the bulbs, with samples lacking DNA amplification excluded from the analysis. Each reaction was repeated three times.

## 5. Conclusions

In this study, a pathogenic fungus was isolated from infected *F. taipaiensis* bulbs from a field in Sichuan, China, and was identified as *F. solani*. To our knowledge, this is the first report of *F. solani* infection in *F. taipaiensis*, which may pose a significant threat to the *F. taipaiensis* industry. The colonization pattern of *F. solani* in the bulb of *F. taipaiensis* was analyzed via microscopic observation. Subsequently, we developed two methods based on LAMP and qPCR for the detection of *F. solani*. These methods were successfully applied to detect the presence of the pathogen in naturally decaying bulbs in the field.

## Figures and Tables

**Table 1:** LAMP and PCR detection and identification technology for *F. solani*. In the column of symptoms, “+” indicates that the bulb was symptomatic and “−“ means that the bulb was asymptomatic. In the column of LAMP results, “+” indicates that the amplicon was detected and “−“ means that no amplicon was obtained.

Sample No.	Sample Source	Sampling Date	Symptoms	LAMP Results	Gene Concentration (pg/g bulb) *
1	Hanzhong	June 2023	−	+	13.00 ± 2.72
2	Hanzhong	June 2023	−	−	0
3	Hanzhong	June 2023	−	−	0
4	Hanzhong	June 2023	−	−	0
5	Hanzhong	June 2023	−	−	0
6	Hanzhong	June 2023	−	−	0
7	Hanzhong	June 2023	−	−	0
8	Hanzhong	June 2023	−	−	0
9	Hanzhong	June 2023	−	−	0
10	Hanzhong	June 2023	−	−	0
11	Hanzhong	June 2023	−	−	0
12	Hanzhong	June 2023	−	−	0
13	Hanzhong	June 2023	−	−	0
14	Hanzhong	June 2023	−	−	0
15	Hanzhong	June 2023	−	−	0
16	Hanzhong	June 2023	−	−	0
17	Shangluo	May 2023	−	−	0
18	Shangluo	May 2023	−	−	0
19	Shangluo	May 2023	−	−	0
20	Shangluo	May 2023	−	−	0
21	Shangluo	May 2023	−	−	0
22	Shangluo	May 2023	−	−	0
23	Shangluo	May 2023	−	−	0
24	Shangluo	May 2023	+	+	985.84 ± 48.62
25	Shangluo	May 2023	−	−	0
26	Shangluo	May 2023	−	−	0
27	Shangluo	May 2023	−	−	0
28	Shangluo	May 2023	−	−	0
29	Shangluo	May 2023	−	−	0
30	Shangluo	May 2023	−	−	0
31	Shangluo	May 2023	−	−	0
32	Shangluo	May 2023	+	+	126.73 ± 6.22
33	Guangyuan	June 2023	−	−	0
34	Guangyuan	June 2023	−	−	0
35	Guangyuan	June 2023	−	−	0
36	Guangyuan	June 2023	−	−	0
37	Guangyuan	June 2023	−	−	0
38	Guangyuan	June 2023	−	−	0
39	Guangyuan	June 2023	−	−	0
40	Guangyuan	June 2023	+	+	51.36 ± 8.26
41	Guangyuan	June 2023	−	−	0
42	Guangyuan	June 2023	−	−	0
43	Guangyuan	June 2023	−	−	0
44	Guangyuan	June 2023	−	−	0
45	Guangyuan	June 2023	−	−	0
46	Guangyuan	June 2023	+	+	22.10 ± 2.96
47	Guangyuan	June 2023	+	+	235.94 ± 5.87
48	Guangyuan	June 2023	−	+	3.58 ± 0.76

* The *acl1* gene concentrations of *F. solani* detected in bulbs of *F. taipaiensis*.

**Table 2:** Primers used in this study.

PCR Type	Gene	Primer Name	Sequence (5′→3′)	Length (bp)	Reference
PCR	ITS	ITS1	TCCGTAGGTGAACCTGCGG	530	[60]
		ITS4	TCCTCCGCTTATTGATATGC		
	*EF-1α*	EF-1	ATGGGTAAGGARGACAAGAC	714	[29]
		EF-2	GGARGTACCAGTSATCATG		
	*ACT*	ACT-512F	ATGTGCAAGGCCGGTTTCGC	269	[61]
		ACT-783R	TACGAGTCCTTCTGGCCCAT		
qPCR	*acl1*	FS-R	GGTCCGCGATGTAAGTTGAA	221	This study
		FS-F	AACCCACAAATCCAGACCAG		
LAMP	*acl1*	FS-F3	CGGAAATCTACCGAGTGCTC	218	This study
		FS-B3	GGGACGTTCTTGAGGAGAGT		
		FS-FIP	CCAGTCGCCCTATGGTCAATCG CGGACCACCGATCCATCT		
		FS-BIP	GATGTCGGTGATGTTGACGCCA TCCTCGTTGGAGGGGTAC		
		FS-LB	AGGCTGAGAAGCTCTTGATCC		

## Data Availability

Data are contained within the article.

## Supplementary Materials

The following supporting information can be downloaded at: https://www.mdpi.com/article/10.3390/plants13162236/s1, Figure S1: The symptoms of bulb rot of *F. taipaiensis* in the field; Figure S2: Design of LAMP primers specific for *F. solani* based on *acl1* sequences; Figure S3: The locations of the specific primers in the *acl1* gene sequence of *F. solani* (GenBank: MW810996); Figure S4: Design of qPCR primers specific for *F. solani* based on *acl1* sequences; Table S1: ITS, *EF-1α*, and *ACT* gene accession numbers for 12 strains used in the construction of the phylogenetic tree of the strain FTA1 and the related *Fusarium* species; Table S2: Specificity tests of the LAMP and PCR primers for *F. solani*.
