# Supplementary material for: Identification of Pathogen Causing Bulb Rot in Fritillaria taipaiensis P. Y. Li and Establishment of Detection Methods
Source: Plants (Basel). 2024 Aug 12;13(16):2236. doi: 10.3390/plants13162236 (PMC11360731; doi:10.3390/plants13162236)
Supplement: Supplementary file 1 [file plants-13-02236-s001.zip › plants-3129812-supplementary.pdf]

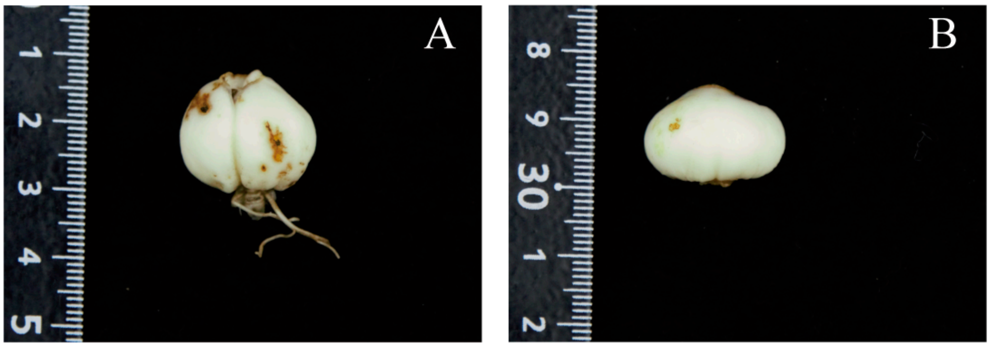

Figure S1. The Symptoms of bulb rot of *F. taipaiensis* in the field. (**A**) and (**B**) Naturally decaying bulbs.

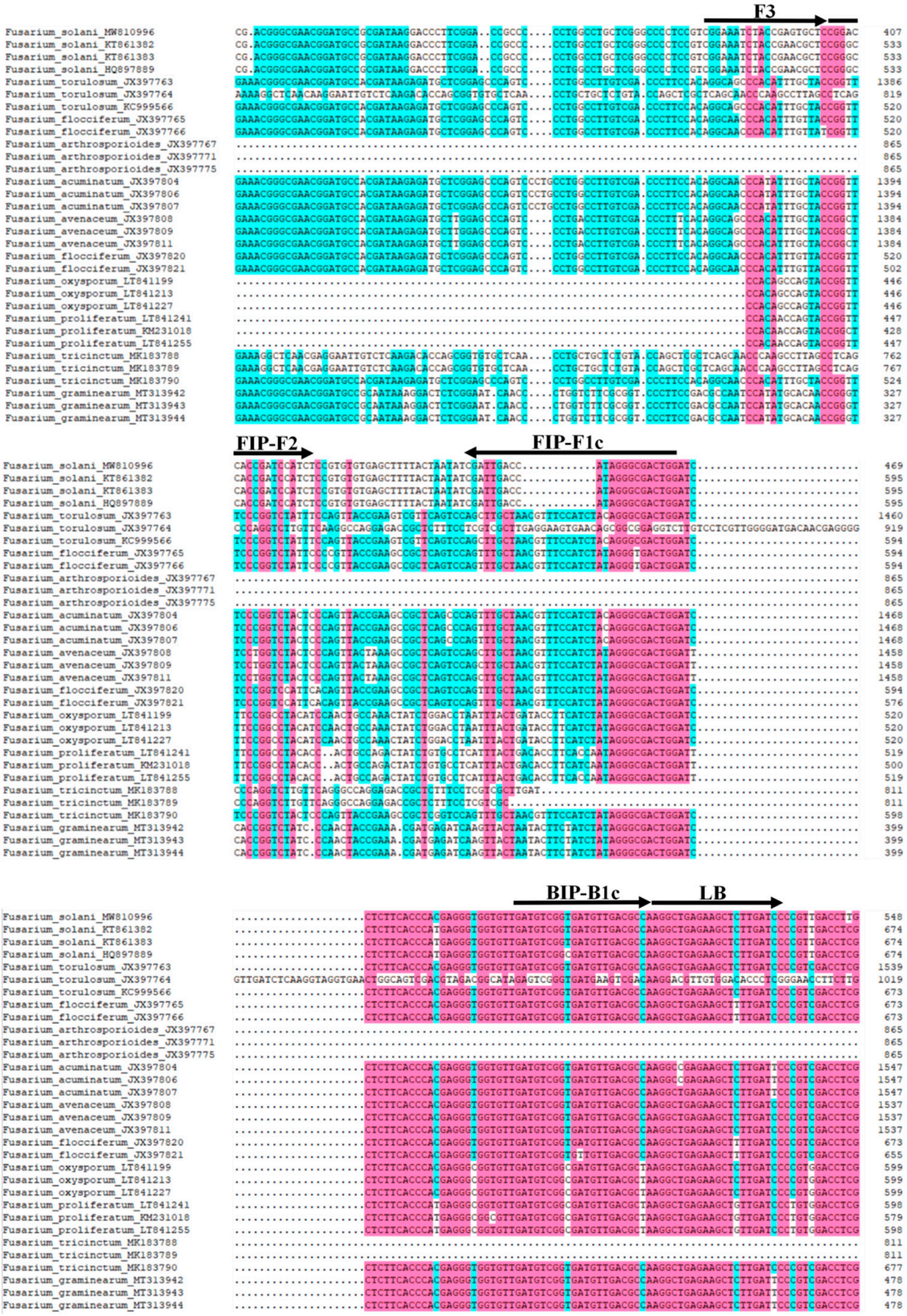

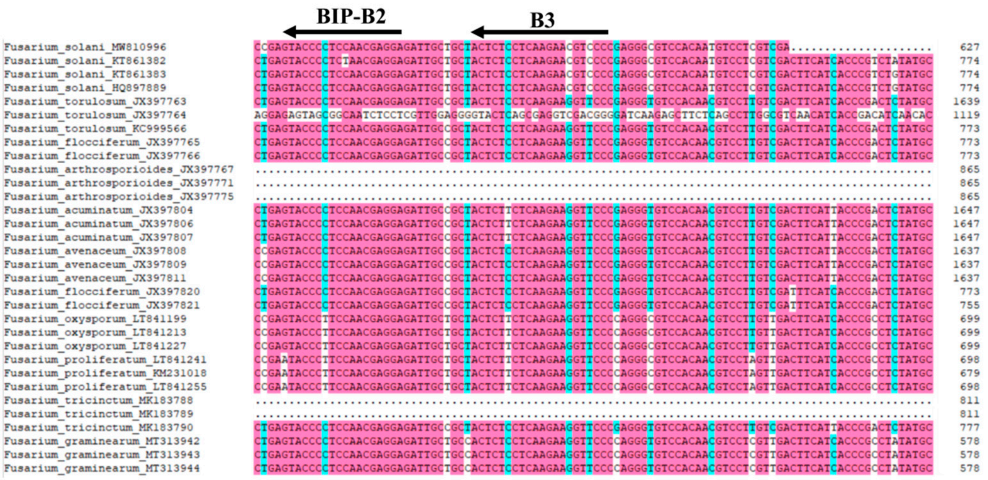

Figure S2. Design of LAMP primers specific for *F. solani* based on *acl1* sequences. Nucleotide sequence alignment of *acl1* sequences from *F. solani* and other *Fusarium* species. Partial sequences of *acl1* and the location of five LAMP primers (F3, B3, FIP (F1c-F2), BIP (B1c-B2), and LB) were shown. Arrows indicate the direction of extension.

321 GCGAACGGAT GCCGCGATAA GGACCCTTCG GACCGCCCCC TGGCCTGCTC GGGCCCCTCC

381 GTCGGAAATC TACCGAGTGC TCCGGACCAC CGATCCATCT CCGTGTGTGA GCTTTTACTA

F3 FIP-F2

441 ATATCGATTG ACCATAGGGC GACTGGATCC TCTTCACCCA CGAGGGTGGT GTTGATGTCG

FIP-F1c

501 GTGATGTTGA CGCCAAGGCT GAGAAGCTCT TGATCCCCGT TGACCTTGCC GAGTACCCCT

BIP-B1c LB

561 CCAACGAGGA GATTGCTGCT ACTCTCCTCA AGAACGTCCC CGAGGGCGTC CACAATGTCC

BIP-B2 B3

621 TCGTCGA

Figure S3. The locations of the specific primers in the acl1 gene sequence of *F. solani* (GenBank: MW810996). The primers F3, B3, LF, FIP (F1c+F2), and BIP (B1c+B2) are indicated by underlined sequences and arrows.

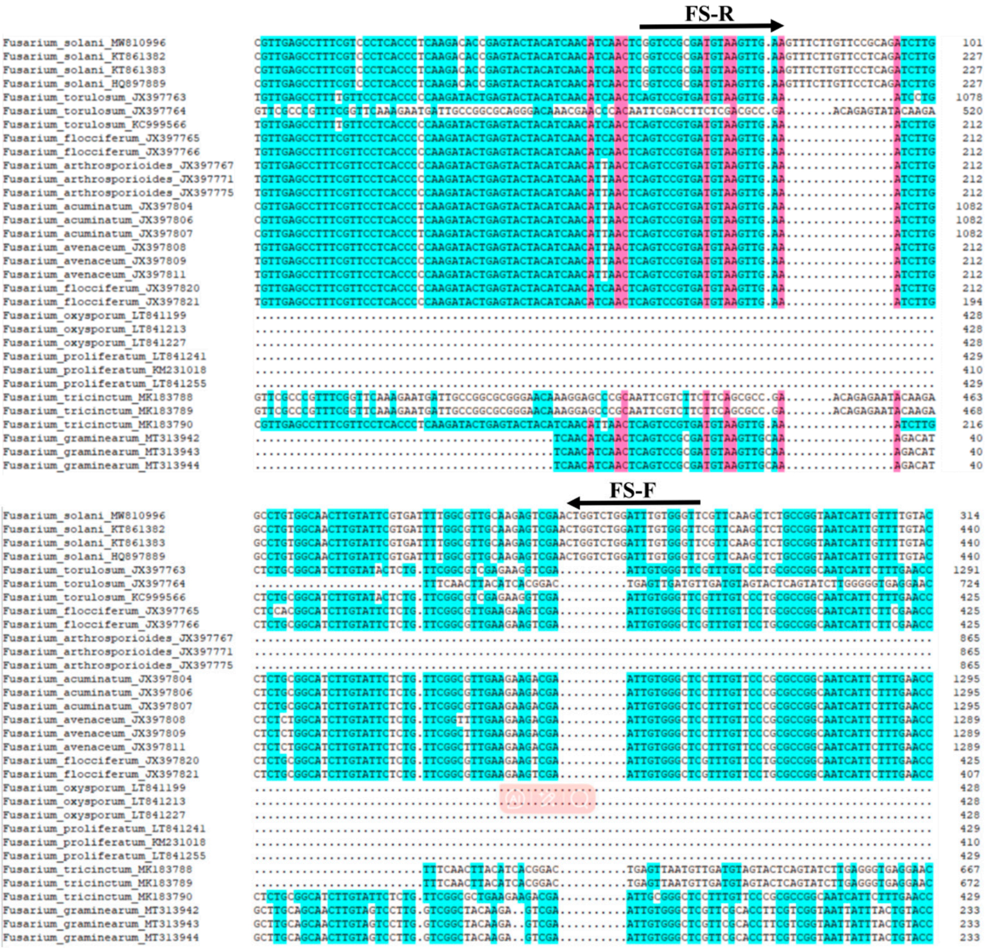

Figure S4. Design of qPCR primers specific for *F. solani* based on *acl1* sequences. Nucleotide sequence alignment of *acl1* sequences from *F. solani* and other *Fusarium* species.

Table S1. ITS, *EF-1α* and *ACT* gene accession numbers for 12 strains used in the construction of the phylogenetic tree of the strain FTA1 and the related *Fusarium* species.

	Species	Strain number	ITS	*EF-1α*	*ACT*
001	***Fusarium solani***	**FTA1**	**PP758636**	**PP766248**	**PP785370**
002	*Fusarium solani*	CBS 102429	KM231808	KM231936	KM231204
003	*Fusarium solani*	CREA OF 897.1.1	OQ255944	OQ274879	OQ249626
004	*Fusarium oxysporum*	CBS 130302	MH865881	MT011000	MT010876
005	*Fusarium oxysporum*	PSD-3	ON927009	ON938187	
006	*Fusarium proliferatum*	CBS 116324	KR071674	KR071731	KU603844
007	*Fusarium ananatum*	CBS 402.97	MH862652	MT010989	KU603802
008	*Fusarium concolor*	CBS 961.87	KR071662	KR071773	KU603850
009	*Fusarium terricola*	CBS 483.94	KU604040	KU711698	KU603797
010	*Fusarium fujikuroi*	CBS 221.76	MW827608	MN534010	KU603834
011	*Fusarium subglutinans*	CBS 747.97	KR071623	MW402150	KU603821
012	*Fusarium ficicrescens*	CBS 125178	KU604031	KU604452	KU603780

Table S2. Specificity tests of the LAMP and PCR primers for *F. solani.*

Species	Strain[a]	Host or source[b]	Region	LMAP	qPCR
Alternaria alternata	FTY1	*Fritillaria taipaiensis* P. Y. Li	Shaanxi, China	–	–
Aspergillus fumigatus	FTK1	*F. taipaiensis*	Shaanxi, China	–	–
A. flavipes	HF	*Schisandra sphenanthera*	Shaanxi, China	–	–
A. tubingensis	G1	*S. sphenanthera*	Shaanxi, China	–	–
A. westerdijkiae	X1	*S. sphenanthera*	Shaanxi, China	–	–
Botrytis cinerea	FTBC	*F. taipaiensis*	Shaanxi, China	–	–
F. solani	FTA1	*F. taipaiensis*	Sichuan, China	+	+
F. proliferatum	BNCC143058	Cotton	U	–	–
F. avenaceum	FTC1	*F. taipaiensis*	Shaanxi, China	–	–
F. culmorum	BNCC143014	Soil	U	–	–
F. redolens	KM	*Atractylodes chinensis*	Shaanxi, China	–	–
F. graminearum	BNCC337560	Root	U	–	–
F. oxysporum	FTB1	*F. taipaiensis*	Shaanxi, China	–	–
F. moniliforme	BNCC195387	U	U	–	–
Mucor circinelloides	FZCG190604	*Aconitum carmichaelii*	Shaanxi, China	–	–

[a] International identification abbreviations: BNCC, BeNa Culture Collection, China;

[b] U, unknown.

[c] Specificity tests for the LAMP and qPCR detection of *F. solani.* +, positive and –, negative.
